# Molecular Evolution and Epidemiological Dynamics of Foot‐and‐Mouth Disease Virus O/ME‐SA/Ind‐2001e Circulating in East Java, Indonesia, in 2022–2025

**DOI:** 10.1155/vmi/6526830

**Published:** 2026-04-20

**Authors:** Zayyin Dinana, Jola Rahmahani, Suwarno, Iwan Sahrial Hamid, Mohammad Anam Al-Arif, Muchammad Yunus, Wiwiek Tyasningsih, Igo Syaiful Ihsan, Aussie Tahta Maharani, Firdausy Kurnia Maulana, Nur Saidah Said, Deka Uli Fahrodi, Fedik Abdul Rantam

**Affiliations:** ^1^ Doctoral Program of Veterinary Science, Faculty of Veterinary Medicine, Universitas Airlangga, Surabaya, Indonesia, unair.ac.id; ^2^ Research Center on Global Emerging and Re–Emerging Infectious Diseases, Institute of Tropical Disease, Universitas Airlangga, Surabaya, Indonesia, unair.ac.id; ^3^ Division of Microbiology, Faculty of Veterinary Medicine, Universitas Airlangga, Surabaya, Indonesia, unair.ac.id; ^4^ Division of Basic Veterinary Science, Faculty of Veterinary Medicine, Universitas Airlangga, Surabaya, Indonesia, unair.ac.id; ^5^ Division of Animal Husbandry, Faculty of Veterinary Medicine, Universitas Airlangga, Surabaya, Indonesia, unair.ac.id; ^6^ Division of Veterinary Parasitology, Faculty of Veterinary Medicine, Universitas Airlangga, Surabaya, Indonesia, unair.ac.id; ^7^ Department Basic of Veterinary Medicine, Faculty of Health, Medicine and Natural Sciences, Universitas Airlangga, Surabaya, Indonesia, unair.ac.id; ^8^ Airlangga Disease Prevention and Research Center, Universitas Airlangga, Surabaya, Indonesia, unair.ac.id; ^9^ Faculty of Animal Husbandry and Fisheries, Universitas Sulawesi Barat, Majene, Indonesia; ^10^ Research Center for Vaccine Technology and Development, Institute of Tropical Disease, Universitas Airlangga, Surabaya, Indonesia, unair.ac.id

**Keywords:** evolution, foot-and-mouth disease virus, Indonesia, phylogeny, VP1

## Abstract

**Background:**

Indonesia has been free from foot‐and‐mouth disease virus (FMDV) for decades, but in April 2022, the first major re‐emerging of FMDV was detected in Gresik, East Java, and later spreading nationwide. To understand how dynamic virus spread occurs in Indonesia, we performed an epidemiological study and molecular evolution analyses.

**Methods:**

A total of 40 FMDV suspected samples collected in 2023–2025 from cattle in East Java, Indonesia. Phylogeny were inferred using the neighbor‐joining method, and Bayesian Markov Chain Monte Carlo (MCMC) was employed to estimate the evolutionary rate and time to the most recent common ancestor (tMRCA) compared with global reference and vaccine strains.

**Results:**

Sanger sequencing was performed for 10 out of 40 positive VP1 genes, and results were confirmed as O/ME‐SA/Ind‐2001e. Amino acid substitutions were identified within the G‐H loop region of the VP1 protein and play an important antigenic site involved in receptor binding and immune recognition. The Bayesian evolutionary analyses suggested that estimated evolutionary rate was 1.1 × 10^−3^ substitutions/site/year and tMRCA was 2012 (95% HPD interval 2008–2025).

**Conclusion:**

Amino acid mutations in G‐H loop region may affect viral infectivity, host tropism, and vaccine match. These findings emphasize the need for continuous molecular surveillance of FMDV in Indonesia to detect emerging variants, understand transmission dynamics, and maintain effective antigenic matching between vaccines and circulating field strains.

## 1. Introduction

Foot‐and‐mouth disease (FMD) is a highly contagious viral disease of cloven‐hoofed livestock especially in cattle with major impacts on animal health, productivity, livelihoods, and trade [1]. The FMD virus (FMDV) serotype O topotypes O/ISA‐1 and O/ISA‐2 circulated in Indonesia prior to 1984 but are now considered extinct and likely introduced into the region in the late 19th century before being gradually replaced by other serotypes and topotypes in subsequent decades of the 20th century [2]. After more than 3 decades of being FMD‐free, Indonesia experienced outbreaks of FMD in East Java in April 2022 caused by a new genotype of serotype O virus, O/ME‐SA/Ind‐2001e, representing a modern and genetically distinct lineage that was detected following extensive FMD surveillance and molecular characterization of outbreak isolates [3–5]. Subsequent investigations and national responses including large vaccination campaigns beginning in late 2024 show that the disease remains a pressing veterinary public‐policy issue in Indonesia.

The disease is caused by the FMDV, a positive‐sense single‐stranded RNA virus belonging to the genus *Aphthovirus* within the family Picornaviridae [1, 6]. FMDV is characterized by its high mutation rate, which contributes to the emergence of multiple antigenic variants and complicates vaccine development and disease control efforts [7]. There are seven immunologically distinct serotypes (O, A, C, Asia‐1, SAT 1, SAT 2, and SAT 3), and infection with one serotype does not confer protective immunity against others [1, 6].

Indonesia presents a unique case in the global FMD landscape. After achieving freedom from the disease in 1986, the country remained officially recognized as FMD‐free without vaccination by the World Organization for Animal Health (WOAH) for several decades [3, 8]. However, FMD re‐emerged in East Java, Indonesia, and quickly spread to multiple provinces, marking a significant setback for national livestock production and trade [5]. Globally, FMD remains endemic in parts of Africa, Middle East, and Asia, with sporadic incursions into previously disease‐free countries [1]. The epidemiology of FMD is complex and influenced by factors such as livestock movement, animal density, wildlife reservoirs, vaccination coverage, and biosecurity practices [9]. Control strategies typically include vaccination, movement restrictions, improved biosecurity, and surveillance, though success varies depending on infrastructure, resources, and political commitment [10].

Molecular analyses of outbreak samples have primarily identified as O/ME–SA/Ind‐2001e lineage, consistent with circulating lineages in Southeast Asia [10]. The presence of hypervariable regions, the G–H loop in the VP1 gene, is considered to play a crucial role in genotype variation, as it is highly associated with mutations and/or recombination events that contribute to viral evolution and antigenic diversity [11]. The evolutionary analyses or evolutionary studies of FMDV have been extensively employed based on the VP1 coding sequence [12, 13], but this has not been done yet in Indonesia. Identification of FMDV serotype as O/ME–SA/Ind‐2001e has been published [3–5]. From 2022 to the present, several FMDV outbreaks still reported in several regions in Indonesia, despite vaccination efforts [3]. The continued discovery of FMDV cases in Indonesia, even though the government’s implementation of a mass vaccination program, has prompted research to focus on the FMD virus genome and host tropism. In this study, we aim to characterize FMDV isolates collected during the period 2022–2025 through evolutionary analysis based on the VP1 gene sequence. The evolutionary patterns and genetic diversity among Indonesian FMDV isolates will be compared with FMDV isolates detected in other countries.

## 2. Materials and Methods

### 2.1. Samples Collection and Time of Research

A total of 40 samples were collected from smallholder cattle farms exhibiting clinical signs of FMD, including fever, depression, hypersalivation, and vesicular lesions in the mouth and feet. The samples were obtained from farms located in Banyuwangi, Pasuruan, Probolinggo, Lumajang, and Gresik, all within East Java Province, Indonesia, during the period of 2023–2025. Vesicle swab was collected and placed into 5 mL sterilized tube containing viral transport medium (MEM, 1% antibiotic and Fungizone). All samples were stored at −80°C freezer in the laboratory until analysis. The 2022 sequence samples were retrieved from DDBJ/EMBL/GenBank databases with accession number PP066254‐PP066270. This study was approved by Ethics Committee at the Faculty of Veterinary Medicine, Universitas Airlangga, Surabaya, Indonesia. The ethics statement is provided in Supporting Figure S1.

### 2.2. FMD Viral Detection

Viral RNA was extracted using a QIAmp Viral RNA Mini Kit according to the manufacturer’s instructions (Qiagen, Hilden, Germany). RNA was eluted with 60 μL of RNase‐DNase‐free water and stored at −80°C. The reverse transcriptase reaction was performed using 5 μL of RNA and 15 μL Taq polymerase reaction mix, 0.7 μL of enzyme mix, and 4.25 μL of RNase‐free water at 65°C for 3 min, followed by 40°C for 1 h (GoScript Reverse Transcriptase, Promega, USA). We used the previously published primer pair of FMDV serotype O in the VP1 gene, forward primer O1C244F (5′‐GCA​GCA​AAA​CAC​ATG​TCA​ACA​CCT​T‐3′) and reverse primers EUR2B52R (5′‐GACATGTCCTC CTGCATCTGGTTGAT‐3′) [14]. The PCR reaction mix was prepared from 5 μL cDNA, 2.5 μL primer forward and reverse, 12.5 μL PCR reagent mix, 5 μL of RNase‐free water (GoTaq Green Master Mix, Promega, USA). The cycles were performed using the following conditions: 5 min at 94°C followed by 35 cycles at 94°C for 60 s, 55.5°C for 60 s, 72°C for 60 s, and 72°C for 10 min. PCR products were analyzed using 2% agarose gel electrophoresis with 1 × Tris‐borate‐ethylenediaminetetraacetic acid buffer (pH 8.3) (Promega, USA) and stained with ethidium bromide (Promega, USA). A total of 5 μL PCR products were placed into the 2% agarose wells. DNA fragments from the positive samples were visualized using the Gel Doc device (Axygen, Corning, USA) under ultraviolet light.

### 2.3. Nucleotide Sequencing and Phylogenetic Analysis

The sequences were determined directly from the PCR products with a BigDye terminator Version 3.1 cycle sequencing kit using an Applied Biosystems 3500XL Genetic Analyzer (Applied Biosystems, Waltham, MA, United States). Nucleotide sequence comparisons were carried out with the references retrieved from GenBank by using the BLAST program (https://blast.ncbi.nlm.nih.gov/) with the sequences of FMD serotype O. Phylogenetic trees were constructed using Molecular Evolutionary Genetic Analysis (MEGA) Version 12.0 with reference sequences retrieved from the DDBJ/EMBL/GenBank databases using the neighbor‐joining method [15]. Alignments were performed using the CLUSTALW MEGA 12, and phylogenetic trees were constructed by the neighbor‐joining method. To confirm the reliability of phylogenetic tree analysis, bootstrap resampling and reconstruction were carried out 1000 times [15].

### 2.4. Bayesian Evolutionary Analysis Using Beast

Estimation time of most recent common ancestor (tMRCA) was determined for the FMD serotype O using the Bayesian Markov Chain Monte Carlo (MCMC) approach as implemented in BEAST Version 1.8.1, and convergence and mixing of the MCMC chains were assessed using Tracer v1.6. All key parameters, including posterior, likelihood, clock rate, strict clock, relaxed clock, and coalescent exponential growth models, showed effective sample size (ESS) values greater than 200, indicating adequate sampling [16]. The dataset for these analyses is comprised of the coding genome sequences of 781 FMDVs circulating in the world during the last 75 years (from 1950 to 2025). The models which are used for BEAST analyses used the GTR + I + G substitution model. MCMC runs were carried out for 500 million generations and evaluated using Tracer Version 1.6 [16]. Only parameters with an ESS of > 200 were accepted. The maximum clade credibility (MCC) trees were annotated with the tree annotator and viewed with FigTree Version 1.4. The statistical uncertainties were summarized in the 95% highest probability density (HPD) intervals [16].

### 2.5. Nucleotide Sequence Accession Numbers

All the nucleotide sequences of the 10 positive VP1 genes were confirmed as O/ME‐SA/Ind‐2001e which has been deposited in the DDJB/GenBank/EMBL database under the accession numbers LC907064–LC907073.

## 3. Results

### 3.1. Nucleotide and Amino Acid Sequence

**FIGURE 1:**
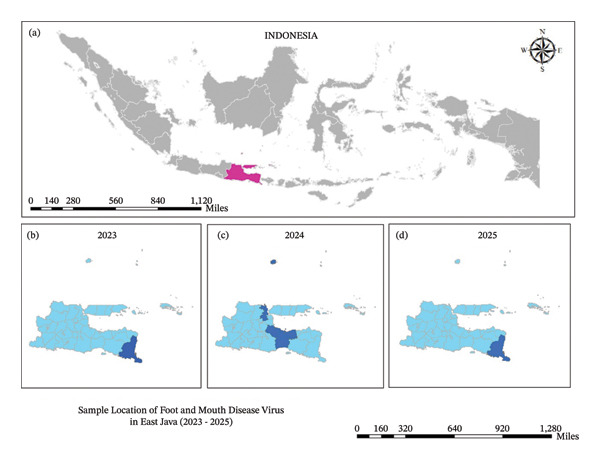
Geographical locations of sample areas of foot and mouth disease virus in East Java, Indonesia, in 2023–2025. (a) Map of Indonesia and the red color indicate East Java Province, (b) dark blue indicates Banyuwangi Regency in 2023, (c) dark blue indicates the districts of Gresik, Pasuruan, Probolinggo, Lumajang in 2024, and (d) dark blue indicates Banyuwangi Regency in 2025.

Out of the 40 representative samples, 10 were successfully amplified by PCR and subsequently sequenced for the VP1 gene, and RNA quality metrics are provided in Supporting Figure S2. In our previous report, in 2022, all sequenced samples confirmed as O serotype, Middle East–South Asia (ME‐SA) topotype, and clustered in into sublineage Ind‐2001e. Figure 1 shows the sampling locations in East Java, Indonesia, in 2023 until 2025.

To perform genetic analysis of Indonesian FMDV strains, we conducted nucleotide and amino acid sequence analysis, focusing on multiple alignment analysis of the VP1 gene associated with immunogenicity. The G‐H loop has critical positions at 135–160 and (43, 44, 144, 148, 149, 154, 208) that are responsible for the antigenic heterogeneity of the VP1 gene. Comparative analysis of nucleotide sequence of three vaccine strains AJ251477 O1/Manisa/Turkey/69, AY593823 O/Manisa/87, and FN594747 O/Manisa/Netherlands revealed variations at three positions (Table 1). Based on the nucleotide mutation, four amino acid substitutions were found in the antigenic sites at D138E, T140A, and A156T based on G‐H loop site (Table 2). Only samples PP066267 and PP066268 have substitutions at position K135Q. The other amino acid positions of VP1 gene (43, 44, 144, 148, 149, 154, 208) that also play as a critical for antigenic sites in this study indicated none substitution. Therefore, the amino acid differences in the antigenic sites between our strain and the vaccine strain may contribute to broad antigenic coverage.

**TABLE 1:** Nucleotide residue in G‐H loop region of VP1 gene of the FMDV isolates in this study compared with vaccine strain. The black box indicates the main variable site selected in the comparison.



**TABLE 2:** Amino acid residue in the G‐H loop region of the VP1 gene of the FMDV isolates in this study compared with the vaccine strain. The black box indicates the main variable site selected in the comparison.



### 3.2. Phylogenetic Tree Analysis

**FIGURE 2:**
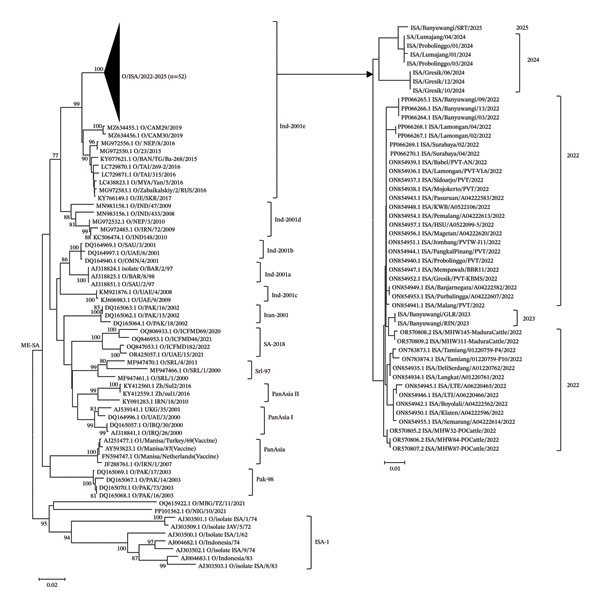
Phylogenetic tree analysis of VP1 gene sequences. A total of 10 samples were detected in this study in 2023–2025 (indicated by black bold font). The VP1 genes of 42 Indonesian and 58 reference FMDV were retrieved from the GenBank database. The genetic distance is indicated at the bottom, and the percent bootstrap support is indicated by the value at each node when the value was 70% or larger.

Phylogenetic tree analysis was performed based on the nucleotide sequence of FMDV VP1 gene. The dynamic serotype of Indonesian FMDV has been detected, and our phylogenetic tree analysis incorporated sequences from the previous Indonesian published strain and available strain of O serotype in the DNA database. To elucidate the relationship between strains found in Indonesia during 2022–2025 and the other reference from database, we analyzed the nucleotide sequences carrying O/ME‐SA comparing with ISA‐1 genotype (Figure 2).

The phylogenetic trees showed that the branch topology of O/ME‐SA in Indonesia detected during 2022–2025 fell within the same monophyletic lineage. The sequence showed high homology identity of 99.2% among the Indonesian isolate (Figure 2). The Indonesian samples clustered in O/ME‐SA/Ind‐2001e with strains from Southeast Asia and has nucleotide sequence identity ranging from 95% to 95.5% (Figure 2). Interestingly, the highest nucleotide and amino acid identity (94.9%) and (95.5%) were found from Cambodia detected in 2019 (Figure 2).

### 3.3. Bayesian Evolutionary Analysis

**FIGURE 3:**
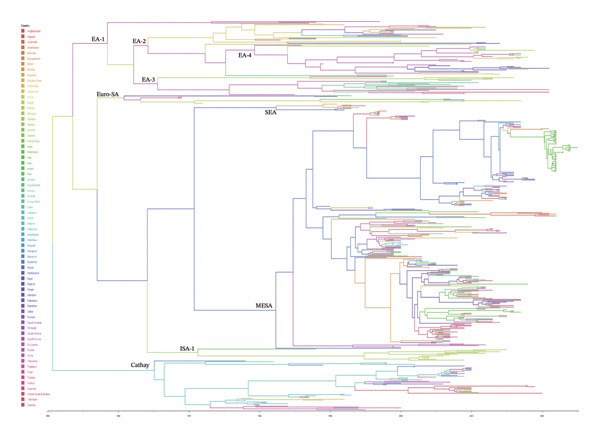
Simplified maximum clade credibility (MCC) drawn from representative sequences of VP1 genes O serotype FDMV. The MCC tree was constructed using a Bayesian MCMC analysis framework with a strict clock model. 706 reference sequences were obtained from the GenBank database. The major lineage has been collapsed for simplicity.

VP1 sequences from this result were analyzed to reconstruct the evolutionary dynamics of these FMDV in Indonesia. To understand the origin and emergence timeline for FMDV O/ME–SA/Ind‐2001e, we performed Bayesian evolutionary analysis on the Indonesian VP1 gene (Figure 3). The analysis of the sequence data over time from the GenBank database enabled the estimation of substitution rates and the tMRCA for the FMDV O serotype. MCC trees were constructed using the Bayesian MCMC framework.

The VP1 gene coding region contains 781 sequences of FMDVs collected during the past 75 years (1950–2025) from the GenBank Database. The estimated time of tMRCA for the VP1 gene in the sublineage O/ME–SA/Ind‐2001e MCC was estimated in 2012 with a predicted tMRCA 95% HPD interval ranging from 2008 to 2025. The estimated evolution rate of FMDV was 1.1 × 10^−3^ (95% HPD interval 1.01 × 10^−3^–1.19 × 10^−3^) (nt substitutions/site/year) (Figure 3). Bayesian evolutionary analysis of the VP1 gene of FMDV isolates from Indonesia in 2022–2025 in the sublineage O/ME–SA/Ind‐2001e parameter estimation based on new data using Bayes’ theorem shows that FMDV appears to have evolved based on population expansion.

## 4. Discussion

In Indonesia, the first FMDV case was reported in April 2022 in Lamongan Regency, East Java, after several decades of being FMDV‐free since 1986 [3]. Previously, we reported an outbreak case in 2022, and the results revealed that this FMDV subtype was included in O/ME‐SA/Ind‐2001e as published [5]. The O/ME‐SA/Ind‐2001e was similar to strains circulating in India, Bangladesh, Myanmar, and Thailand with more than 94% nucleotide identity [17–20]. This trend suggested that the virus spread through trade in animals or livestock products from areas where FMD is still endemic [1].

The genetic diversity of FMDV generates an increasing amount of nucleotide and amino acid sequence information, requiring special handling of the data and the origins of genetic diversity [6, 7]. In this study, the 1D gene, which encodes the VP1 structural protein, is the most variable polyprotein gene [6]. This capsid protein is an immunogenic target for neutralizing antibodies, and variation occurs at specific amino acid sites within the G‐H loop (residues 135–160) [11]. In the G‐H loop of the VP1 coding gene, several amino acid mutations were found in comparison with the vaccine strain [10, 11]. The amino acid substitution is consistent with our previous research findings, occurring at position D138E, T140A, A156T, and only K135Q detected from Lamongan [5]. Residual amino acid variations at T140A, A156T, and A158T have also been reported in Madura cattle and Ongole Grade cattle in Indonesia [4]. Several amino acid substitutions S134A, S137T, T139G, G141 S, and A156T that are found in Egyptian FMDV strains were compared with the vaccine strains, responsible for antigenic heterogeneity [21, 22]. Specific amino acid sites in the G‐H loop region are responsible for viral adaptation and interactions of the viruses to get an escape mutant by the immune systems of vaccinated hosts [22, 23].

The genetic diversity among topotypes highlights the important role of structural variation accumulated throughout the viral genome in host tropism divergence [12]. The evolution of FMDV occurs mainly through the selection to the point mutation or recombination in the antigenic site of VP1 gene [23, 24]. The estimation of FMDV mutation rate is around 10^−3^ to 10^−5^ per nucleotide during genome synthesis and replication cycle, indicating that approximately one mutation appears in each new copy [12, 13]. A time‐scaled Bayesian phylogenetic tree was constructed to examine the evolution of the FMDVs [25, 26].

A time‐scaled Bayesian phylogenetic tree was constructed to examine the evolution of the FMDV O serotype [21]. The evolutionary rate of the VP1 gene in this study was estimated to be 1.1 × 10^−3^ nucleotide substitutions/site/year. This result was similar to those of the VP1 gene O/ME–SA/Ind‐2001e with 6.737 × 10^−3^ substitutions/site/year, O/ME–SA/Ind‐2001 with 1.46 × 10^−3^, and global FMDV serotypes O with 6.338 × 10^−3^ substitutions/site/year [11, 25, 26]. These results align with the evolutionary rates typical of many RNA viruses, which evolve at a rate of approximately 1 × 10^−3^ nucleotide substitutions/site/year, which is parent viruses spread regionally within 1–3 years, whereas the ancestral virus takes 3–5 years to propagate [13, 26, 27]. It is essential to monitor the evolutionary rates of FMDV to identify trends in lineage evolution.

The tMRCA analysis predicts the rate of transmission of FMDV O/ME‐SA/Ind‐2001e [25]. The tMRCA of the VP1 gene in this study was estimated to be in 2012 (95% HPD interval 2008–2025). Monitoring of genotype diversity in India revealed that the predicted tMRCA for the O/ME‐SA/Ind‐2001e lineage was estimated at 2007 (95% HPD interval: 2003–2011), whereas for the O/ME‐SA/Ind‐2001 lineage, it was estimated at 2013 (95% HPD interval: 18–30 years) [13, 27]. Globally, the tMRCA of FMDV serotype O was 481 (95% HPD interval 1932–2007) years ago [26]. In this study, FMDV O/ME‐SA/Ind‐2001e appears in Indonesia regarding a recent tMRCA estimated between late 2021 and early 2022 across Southeast Asian isolates and suggests a rapid cross‐border spread, consistent with increased animal movement or trade.

Continuous molecular surveillance of FMDV across Southeast Asia is essential for monitoring, control, and prevention [1]. New variants may frequently arise that differ antigenically or biologically from existing strains [6]. Molecular monitoring also provides valuable insights into cross‐border transmission dynamics, allowing researchers to trace the geographic movement and evolutionary relationships of viral lineages between countries [10]. Furthermore, the continuous comparison of field isolates with vaccine strains ensures that vaccination programs remain effective by maintaining close antigenic matching [24]. Continuous molecular surveillance is necessary to identify the risk of FMDV outbreaks from new or imported strains that could spread undetected, leading to outbreaks and reduced vaccine match [28].

## 5. Conclusions

The recent identification of the O/ME‐SA/Ind‐2001e lineage highlights the ongoing spread of FMDV in South Asia, with subsequent reports in several Southeast Asian countries. Notably, FMD was first detected in Indonesia in 2022. Molecular characterization of the VP1 coding region revealed several amino acid substitutions in the G‐H loop site. The G‐H loop is a critical antigenic site responsible for receptor binding and recognition by the immune system. Amino acid substitutions are associated with viral adaptation, altered cell receptor interactions, and potential immune escape, which may contribute to effectiveness, host tropism, and vaccine match. The evolutionary rate of the VP1 gene is driven by point mutations and selection pressure at antigenic sites. The tMRCA analysis suggests that the virus underwent recent regional diversification and transmission before its detection in Indonesia. Overall, these findings highlight the importance of continuous molecular surveillance of FMDV in Southeast Asia, especially in Indonesia, to detect emerging variants, understand cross‐border transmission dynamics, and ensure that vaccine strains remain antigenically matched to field viruses. This monitoring is essential to assess the prevalence and genetic diversity of FMDV and to evaluate the efficacy of FMDV vaccines.

## Funding

No funding was received for this manuscript.

## Conflicts of Interest

The authors declare no conflicts of interest.

## Supporting Information

Supporting Table S1 provides additional information regarding the ethical statement obtained from the Faculty of Veterinary Medicine, Universitas Airlangga, with the number: 1.KEH.096.07.2024.

Supporting Table S2 provides information on RNA quality assessment measured using a NanoDrop Lite Plus Spectrophotometer.

## Supporting information


**Supporting Information** Additional supporting information can be found online in the Supporting Information section.

## Data Availability

The data that support the findings of this study are available from the corresponding author upon reasonable request.
