# Supplementary material for: Molecular Evolution and Epidemiological Dynamics of Foot‐and‐Mouth Disease Virus O/ME‐SA/Ind‐2001e Circulating in East Java, Indonesia, in 2022–2025
Source: Vet Med Int. 2026 Apr 20;2026:6526830. doi: 10.1155/vmi/6526830 (PMC13095850; doi:10.1155/vmi/6526830)
Supplement: Supplementary file 1 — Supporting Information Additional supporting information can be found online in the Supporting Information section. [file VMI-2026-6526830-s001.docx]

**Supplementary Table S1. Ethical approval**

**
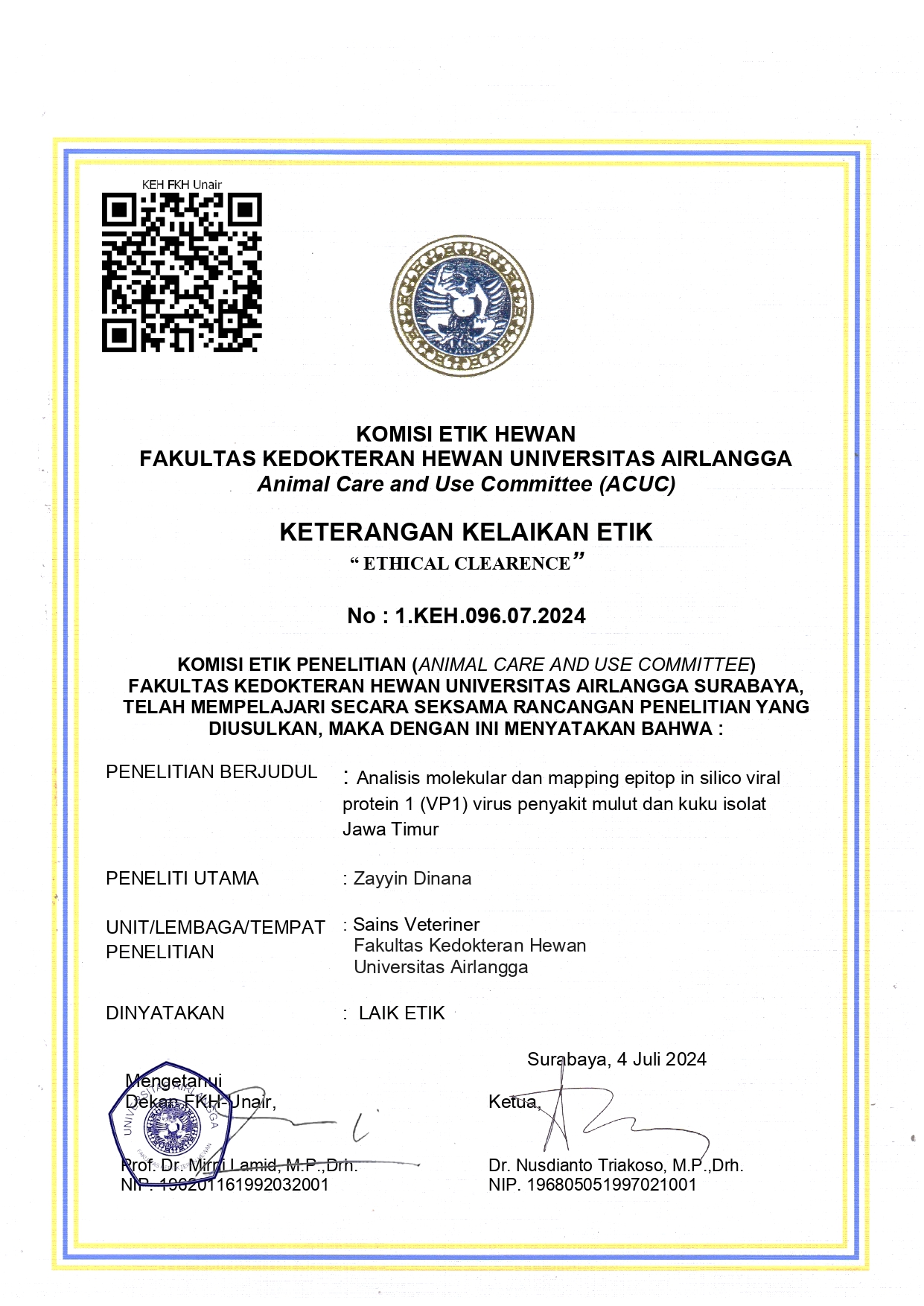
**

**Supplementary Table S2. RNA quality measurement from 10 positive samples using NanoDrop Lite Plus spectrophotometer**

| **No** | **Sample ID** | **Nanodrop Result** |
| --- | --- | --- |
| 1. | ISA/Banyuwangi/GLR/2023 | 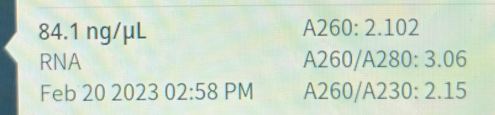 |
| 2. | ISA/Banyuwangi/RIN/2023 | 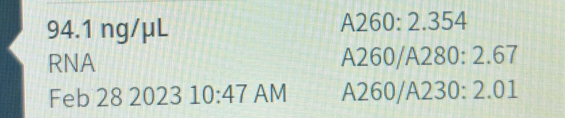 |
| 3. | ISA/Lumajang/01/2024 | 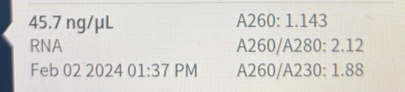 |
| 4. | ISA/Lumajang/04/2024 | 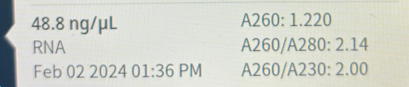 |
| 5. | ISA/Probolinggo/01/2024 | 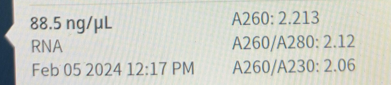 |
| 6. | ISA/Probolinggo/03/2024 | 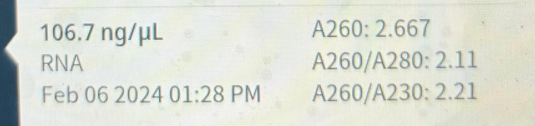 |
| 7. | ISA/Gresik/06/2024 | 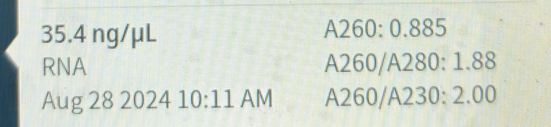 |
| 8. | ISA/Gresik/10/2024 | 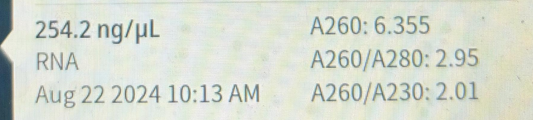 |
| 9. | ISA/Gresik/12/2024 | 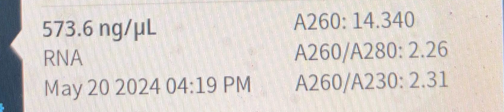 |
| 10. | ISA/Banyuwangi/STR/2025 | 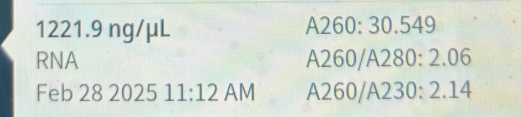 |
